# Whole-body diffusion-weighted MRI of normal lymph nodes: prospective apparent diffusion coefficient histogram and nodal distribution analysis in a healthy cohort

**DOI:** 10.1186/s40644-021-00432-4

**Published:** 2021-11-27

**Authors:** Ricardo Donners, Raphael Shih Zhu Yiin, Matthew Blackledge, Dow-Mu Koh

**Affiliations:** 1 Department of Diagnostic Radiolog, The Royal Marsden NHS Foundation Trust, Downs Road, Sutton, London, Surrey SM2 5PT UK; 2 Department of Diagnostic Radiology, Changi General Hospital, 2 Simei St 3, Singapore, 529889 Singapore; 3 Institute of Cancer Research, 15 Cotswold Road, Sutton, London, SM2 5NG UK; 4 Department of Diagnostic Radiology, Institute of Cancer Research and The Royal Marsden NHS, Foundation Trust, Downs Road, Sutton, London, Surrey SM2 5PT UK

**Keywords:** Whole-body MRI, Diffusion-weighted MRI, Lymph nodes, Histogram

## Abstract

**Background:**

Whole body DWI (WB-DWI) enables the identification of lymph nodes for disease evaluation. However, quantitative data of benign lymph nodes across the body are lacking to allow meaningful comparison of diseased states. We evaluated apparent diffusion coefficient (ADC) histogram parameters of all visible lymph nodes in healthy volunteers on WB-DWI and compared differences in nodal ADC values between anatomical regions.

**Methods:**

WB-DWI was performed on a 1.5 T MR system in 20 healthy volunteers (7 female, 13 male, mean age 35 years). The b900 images were evaluated by two radiologists and all visible nodes from the neck to groin areas were segmented and individual nodal median ADC recorded. All segmented nodes in a patient were summated to generate the total nodal volume. Descriptors of the global ADC histogram, derived from individual node median ADCs, including mean, median, skewness and kurtosis were obtained for the global volume and each nodal region per patient. ADC values between nodal regions were compared using one-way ANOVA with Bonferroni post hoc tests and a *p*-value ≤0.05 was deemed statistically significant.

**Results:**

One thousand sixty-seven lymph nodes were analyzed. The global mean and median ADC of all lymph nodes were 1.12 ± 0.27 (10^− 3^ mm^2^/s) and 1.09 (10^− 3^ mm^2^/s). The average median ADC skewness was 0.25 ± 0.02 and average median ADC kurtosis was 0.34 ± 0.04. The ADC values of intrathoracic, portal and retroperitoneal nodes were significantly higher (1.53 × 10^− 3^, 1.75 × 10^− 3^ and 1.58 × 10^− 3^ mm^2^/s respectively) than in other regions. Intrathoracic, portal and mesenteric nodes were relatively uncommon, accounting for only 3% of the total nodes segmented.

**Conclusions:**

The global mean and median ADC of all lymph nodes were 1.12 ± 0.27 (10^− 3^ mm^2^/s) and 1.09 (10^− 3^ mm^2^/s). Intrathoracic, portal and retroperitoneal nodes display significantly higher ADCs. Normal intrathoracic, portal and mesenteric nodes are infrequently visualized on WB-DWI of healthy individuals.

**Trial registration:**

Royal Marsden Hospital committee for clinical research registration number 09/H0801/86, 19.10.2009.

## Background

Lymph nodes are commonly affected by malignant disease and their involvement impacts patient management [1]. Consequently, early detection and accurate staging of malignant lymph nodes through imaging is of paramount clinical importance. Their evaluation by conventional CT or MRI relies on size thresholds and subjective, non-quantitative assessment of morphologic features to discriminate between diseased and normal states. This method was shown to have limited diagnostic accuracy [2].

Whole body diffusion weighted MRI (WB-DWI) has emerged as a functional imaging technique, which can aid lymph node characterization by providing information on tissue characteristics over a large field of view within reasonable acquisition times – properties, which make it a desirable staging and screening technique [3, 4]. WB-DWI was deemed an alternative to established ^18^FDG PET/CT for lymphoma staging [5]. DWI detects microstructural and cellular alterations in malignant versus benign lymph nodes and can be quantified by calculation of the apparent diffusion coefficient (ADC). The latter was shown to inversely correlate with cellularity, with good measurement repeatability, allowing for tissue characterization [6–9]. Several regional and WB-DWI studies have shown lower ADC values in malignant compared to non-diseased nodes, suggesting the use of ADC as a biomarker for lymph node assessment [10–18]. However, these studies report overlap between malignant and benign states and as a consequence a universal, consistent ADC threshold that can be applied across different cancers to accurately identify malignancy is lacking. One major contributor to this problem is that the published literature on ADC values of benign nodes were derived from patients with pre-existing malignancies. Data on ADC values of normal lymph nodes of a healthy population are lacking. Additionally, there is paucity of data with regards to how nodal ADC values may vary across anatomical sites. Establishment of nodal ADC ranges throughout the body and regional differences in unequivocally normal lymph nodes may facilitate identification of diseased states. Consequently, the purpose of this study is to determine the WB-DWI derived ADC values of normal lymph nodes of healthy individuals and compare them between anatomical regions to establish healthy, nodal ADC ranges.

## Methods

### Study design and study population

Approval for this prospective, non-randomized study performed at a single institution was obtained from the local institutional research and ethics committee. Twenty healthy adult volunteers were recruited and written informed consent was obtained. Exclusion criteria were previous surgery, known or prior malignancy, acute inflammation or infection within 4 weeks prior to scanning and current use of short- or long-term medication.

### Whole-body MR imaging protocol

Free-breathing axial single-shot, spin-echo, echo planar imaging WB-DWI was performed on a 1.5 T MRI scanner (MAGNETOM Avanto, Siemens Healthineers, Erlangen, Germany) using the following imaging parameters: Matrix = 150 × 144, Partial Fourier = 6/8, TE = 64.8 ms, TR = 14.6 s, Receiver Bandwidth = 1961 Hz/px, b-values = 50 and 900 s/mm^2^, three scan trace weighted diffusion encoding, STIR fat suppression (TI = 180 ms), Slice Thickness = 5 mm, Field of View = 400 × 390 mm^2^. GRAPPA was applied to reduce distortion along the phase-encoding direction (R = 2). The number of signal averages was four. Images were acquired from vertex to midthigh using four consecutive imaging stations comprising of 50 slices each. The acquisition time was approximately 24 min. To facilitate anatomical localization of lymph nodes, breath-hold axial T1-weighted MRI, matched to the same imaging field of view and slice thickness of WB-DWI were acquired using the following imaging parameters: T1/FL2D, matrix = 256 × 105, slice thickness = 5 mm, spacing = 5 mm, TR = 386 ms, TE = 4.8 ms, flip angle = 70°, number of averages = 1.

### Image interpretation and processing

The b900 DW images were reviewed by two radiologists with six and eight years of experience in MRI interpretation, respectively. Open-access software (OsiriX version 56, PixmeoSARL Bernex, Switzerland) was used for analyses and post-processing. Facilitated by the anatomical T1-weighted sequences, all lymph nodes were identified on high b-value images as discrete ovoid structures demonstrating high signal intensity and appeared intermediate to low signal intensity, some with a fatty, hilum on the corresponding T1-weighted images. A volume of interest (VOIs) was generated, encompassing an individual node on b900 images, using a semi-automated segmentation software within OsiriX employing a “GrowCUT” algorithm [19]. This was repeated for every lymph node visible on b900 images. When necessary, VOIs were manually adjusted to the nodal parenchyma. All nodal VOIs of a volunteer were transferred onto the corresponding ADC maps. For each lymph node, the nodal volume (ml) and median ADC (in 10^− 3^ mm^2^/s) were recorded.

In each volunteer, all individual nodal volumes were summated to create the global nodal volume (ml). From this global volume the global ADC (gADC) histogram, based on individual node median ADC values was derived. Histogram descriptors including mean, median, skewness and kurtosis were recorded.

**Fig. 1:**
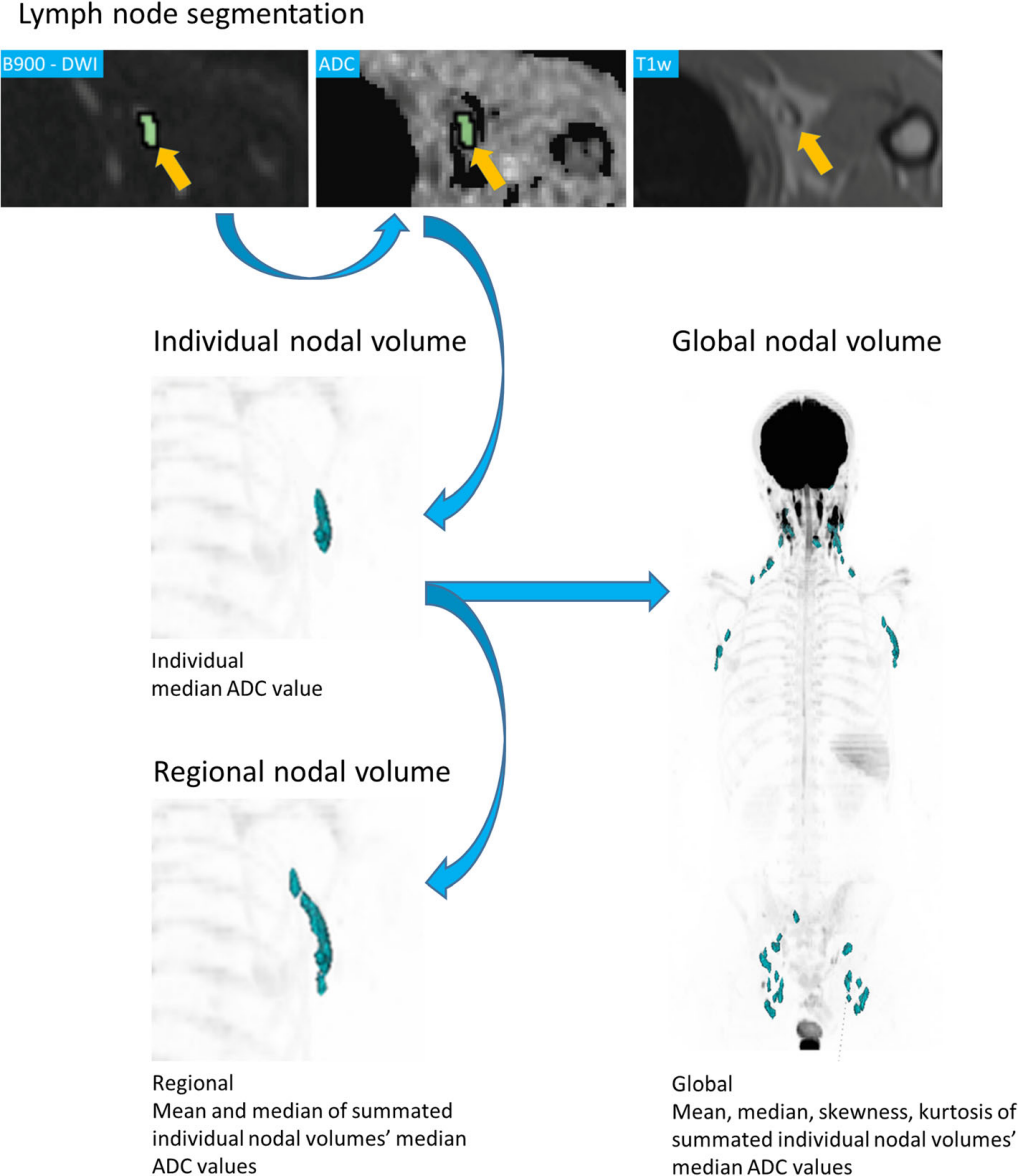
Workflow of nodal volume segmentation and Apparent Diffusion Coefficient (ADC) measurement in a 30-year-old male. A left axillary lymph node is identified on anatomical T1 weighted (T1w) and b900 DWI (arrows) and segmented (green) on b900 images. The resulting volume of interest (VOI) was copied onto the ADC map and individual nodal median ADC derived. Individual nodal volumes were summated to regional (axillary group) and global nodal volumes. Individual nodal median ADC distribution descriptors within these summated volumes (mean, median, skewness, kurtosis) were derived

Additionally, individual nodal volumes were summarized per anatomical site to create regional nodal volumes (ml). From these regional volumes, mean and median of the included individual median nodal ADCs were recorded. The workflow is visualized in Fig. 1.

### Lymph node regions

The lymph nodes were grouped according to ten anatomical sites: occipital, cervical, axillary, intra-thoracic, portal, mesenteric, retroperitoneal, pelvic, inguinal and subcutaneous regions.

The occipital nodes extend from the occiput to the level of the skull base [20]. The cervical region reaches from the skull base to the supra-clavicular region, with reference made to the imaging based nodal classification for metastatic neck adenopathy [21]. The axillary region is defined as the anatomical axillary triangle [22], which only consist of level 1 axillary lymph nodes in our study. The level 2 and 3 axillary lymph nodes (including infraclavicular nodes) were not encountered and hence not evaluated. The intrathoracic group comprises the mediastinal nodal stations defined by the International Association for the Study of Lung cancer (IASLC), including the internal mammary nodes and excluding the supra-clavicular nodes [23]. The portal group summarises nodes along the portal vein, with the inferior limit set at the spleno-portal venous axis, while the mesenteric group is composed of nodes within the bowel mesentery. The retroperitoneal group includes nodes within the retroperitoneal space. The upper limit is set just inferior to the aortic hiatus and the lower limit at the iliac bifurcation. The pelvic group comprises nodes along the anatomic boundaries of the iliac vessels and pelvic sidewalls. The inguinal group consists of nodes immediately above and below the inguinal ligament, as well as deep nodes medial to the femoral vein. Subcutaneous nodes are superficial nodes that are identified within the subcutaneous tissue anywhere in the body and not included in any one of the defined groups.

### Statistical analysis

Statistical analyses were performed using commercially available software (IBM SPSS Statistics Version 22, IBM Corp. Armonk, and SAS JMP, SAS Institute Inc.). The median ADC parameters were compared between nodal regions utilizing one-way analysis of variance (ANOVA) testing with Bonferroni post hoc tests. Normal distribution was assessed using the Shapiro-Wilk-test. A *p*-value ≤0.05 was taken to be statistically significant. The distributions of median ADC and nodal volume are approximated by kernel density estimation using a Gaussian kernel and a bandwidth approximated by Silverman’s rule of thumb [24]. Pearson coefficients were calculated to assess correlation between volunteer age and global ADC.

## Results

Seven women and 13 men were recruited, with a mean age of 34 years (range 21–59 years). In total 1067 nodes were identified and segmented.

### Nodal distribution

**Fig. 2:**
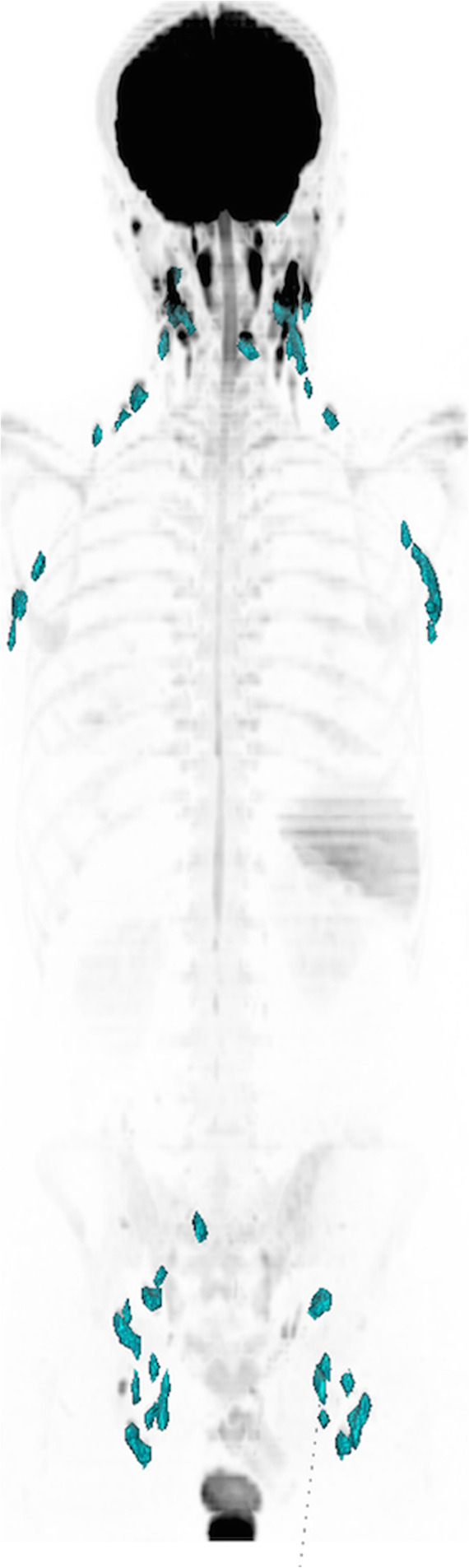
WB-DWI 3D MIP of Nodal Distribution in a 30-year-old male. Typical nodal distribution of an adult volunteer, with majority of nodes in the cervical, axillary and inguinal regions

Lymph node distributions on WB-DWI were observed as follows: occipital (6.6%), cervical (32.2%), axillary (18.4%), intrathoracic (0.9%), mesenteric (1.2%), portal (1%), retroperitoneal (8.9%), pelvic (6%), inguinal (23.9%) and subcutaneous (0.7%). Figure 2 shows the lymph node volumes as identified on a WB-DWI 3D Maximum intensity projection (MIP) of a 30-year-old healthy male.

### Nodal volume

**Fig. 3:**
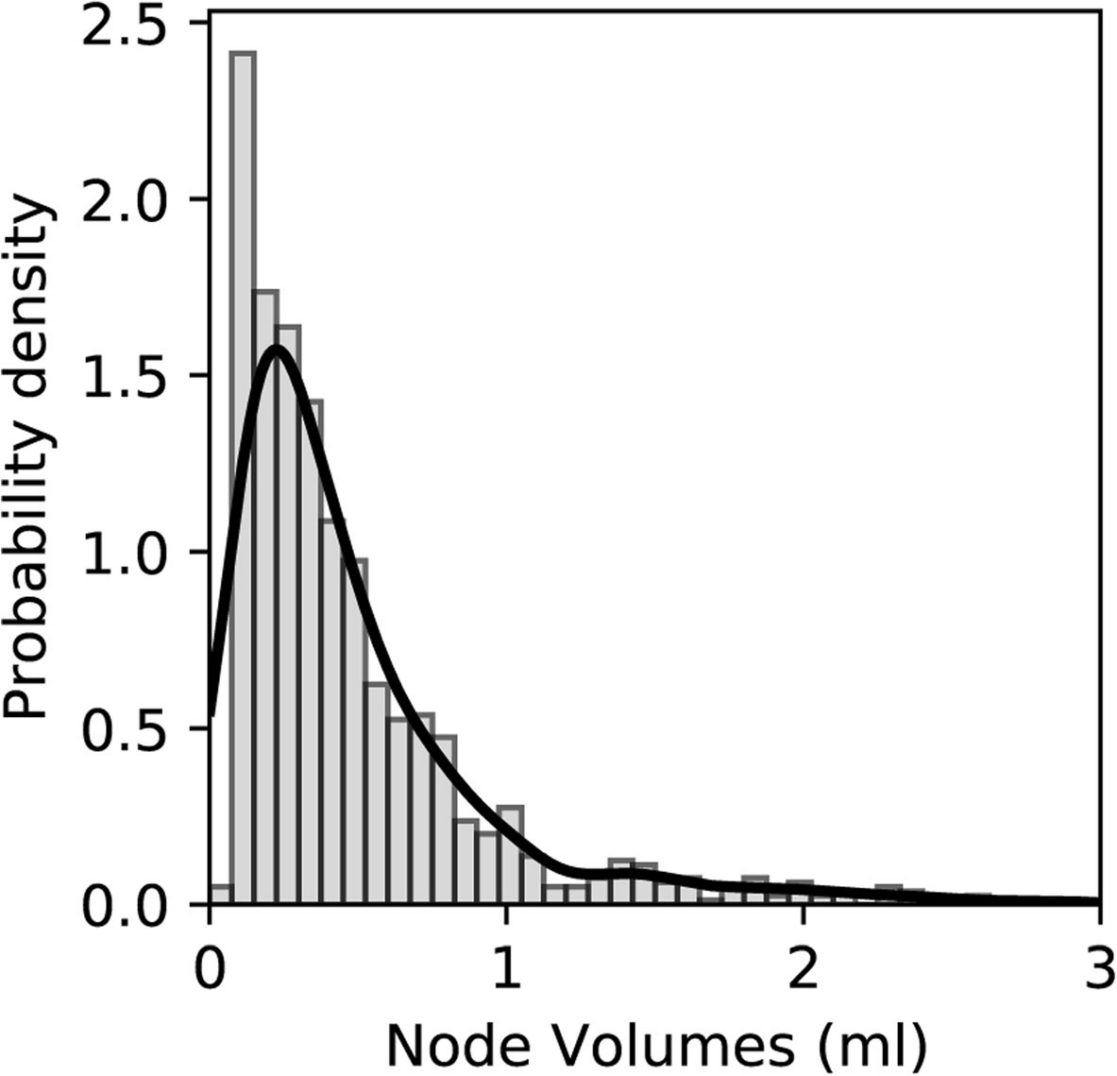
Histogram and kernel density estimation (solid line) showing the distribution of nodal volumes across the entire study cohort including all nodal regions of all participants

The mean ± standard deviation of single lymph node volumes was 0.48 ± 0.44 ml, with a median volume of 0.34 ml and modal volume of 0.12 ml, with values ranging between 0.05 and 2.93 ml. Figure 3 shows the distribution histogram for all nodal volumes across the study cohort.

### ADC histogram parameters of the total measured lymph node volume

**Fig. 4:**
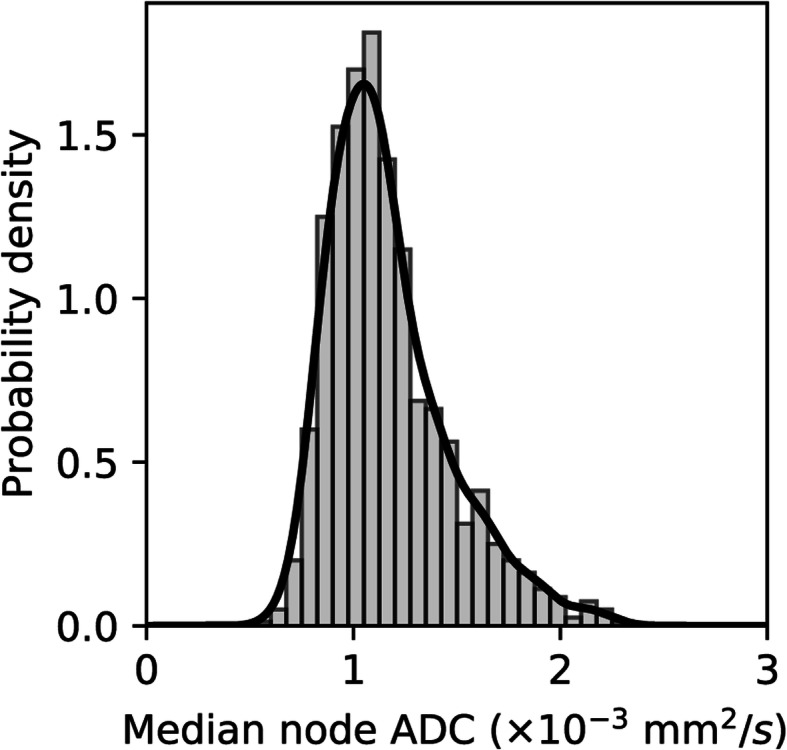
Histogram and kernel density estimation (solid line) showing the distribution of median ADC values of all normal lymph nodes across the entire study cohort including all nodal regions of all participants

The median global ADC values per volunteer are shown in Table 1. The global mean and median of individual nodal median ADC values across all 20 volunteers were 1.12 ± 0.27 (10^− 3^ mm^2^/s) and 1.09 (10^− 3^ mm^2^/s) respectively. Figure 4 shows the histogram distribution curve for the nodal median ADC values of all normal lymph nodes across the study cohort. The average skewness of nodal median ADC was 0.25 ± 0.02; and the kurtosis was 0.34 ± 0.04. No differences between genders were observed (*p* > 0.05). Pearson correlation showed no significant correlation between patient age and global mean/median nodal median ADC (*p* > 0.348, Pearson correlation = − 0.222).

**Table 1:** Data table showing the nodal frequency, mean nodal volume (ml) and median global ADC values (10^− 3^ mm^2^/s) of the 20 volunteers. SD = standard deviation

Volunteers	Number of nodes	Mean Nodal Volume in ml	SD	Median ADC x10^**3**^mm/s^**2**^
**1**	**62**	**0.50**	**0.45**	**0.99**
**2**	**51**	**0.50**	**0.37**	**1.19**
**3**	**26**	**0.54**	**0.58**	**1.16**
**4**	**30**	**0.57**	**0.48**	**1.16**
**5**	**40**	**0.48**	**0.52**	**1.00**
**6**	**76**	**0.77**	**0.59**	**0.91**
**7**	**70**	**0.51**	**0.34**	**1.25**
**8**	**44**	**0.47**	**0.39**	**1.16**
**9**	**69**	**0.37**	**0.32**	**1.14**
**10**	**60**	**0.65**	**0.70**	**1.15**
**11**	**57**	**0.51**	**0.55**	**1.26**
**12**	**62**	**0.48**	**0.31**	**1.03**
**13**	**41**	**0.70**	**0.67**	**1.22**
**14**	**47**	**0.33**	**0.19**	**1.03**
**15**	**61**	**0.46**	**0.40**	**1.00**
**16**	**54**	**0.74**	**0.57**	**1.02**
**17**	**55**	**0.33**	**0.23**	**1.00**
**18**	**57**	**0.57**	**0.41**	**0.90**
**19**	**54**	**0.64**	**0.69**	**1.14**
**20**	**51**	**1.17**	**0.77**	**0.92**

### Comparison across nodal regions

The nodal frequency, mean nodal volumes and median ADC values per anatomical region across the study cohort are shown in Table 2.

**Table 2:** Data table showing the nodal frequency, mean nodal volume (ml) and median ADC values (10^− 3^ mm^2^/s) per nodal region. SD = standard deviation, 95%-CI = confidence interval

Nodal region	Node count	Mean nodal volume in ml	SD	Median ADC x10^**3**^mm/s^**2**^	95% CI
**All nodes**	**1067**	**0.48**	**0.45**	**1.11**	**1.09–1.12**
**Cervical**	**344**	**0.36**	**0.15**	**1.14**	**1.10–1.16**
**Inguinal**	**255**	**0.68**	**0.54**	**0.99**	**0.97–1.01**
**Axilla**	**196**	**0.61**	**0.54**	**1.06**	**1.03–1.08**
**Retroperitoneal**	**95**	**0.32**	**0.25**	**1.61**	**1.53–1.67**
**Pelvic**	**65**	**0.50**	**0.41**	**1.17**	**1.12–1.29**
**Occipital**	**70**	**0.32**	**0.21**	**1.14**	**1.12–1.21**
**Mesenteric**	**13**	**0.46**	**0.25**	**1.18**	**1.08–1.55**
**Portal**	**11**	**0.39**	**0.26**	**1.58**	**1.33–1.66**
**Intrathoracic**	**10**	**0.51**	**0.57**	**1.75**	**1.40–1.92**
**Subcutaneous**	**8**	**0.21**	**0.07**	**0.97**	**0.84–1.41**

**Fig. 5:**
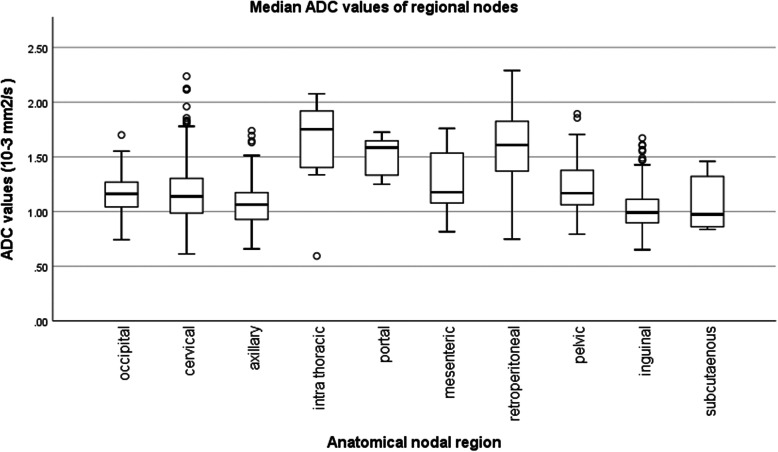
Box-and-whiskers plot of Median ADC values of the Nodal Regions. This box plot shows the distribution of median nodal ADC value across all nodal regions, such that the black line within the box indicates the median of the median ADCs, accompanied by the numerical value on the side. The upper and lower whiskers set the max and min median ADC values respectively in each region

Figure 5 shows the box-and-whiskers plot of nodal median ADC values according to the nodal regions defined in our study.

The regional lymph node median ADC values ranged from 0.98 to 1.18 (10^− 3^ mm^2^/s) for the cervical, inguinal, axillary, occipital, mesenteric, pelvic and subcutaneous nodal groups, comprising 88% of all included lymph nodes. Compared with all other nodal regions, retroperitoneal and intrathoracic nodes showed significantly higher nodal median ADC (1.61 × 10^− 3^ and 1.75 × 10^− 3^ mm^2^/s respectively, *p* < 0.001). Portal nodes also showed significantly higher median ADC (1.58 × 10^− 3^ mm^2^/s) when compared with the regions of high nodal frequency, but not when compared with the mesenteric region (*p* = 0.173).

Among the three most common nodal groups (cervical, axillary and inguinal), each containing more than 190 evaluated nodes in this study, cervical nodes displayed higher median ADC values than axillary or inguinal nodes (p < 0.001). By contrast, intrathoracic, portal and mesenteric nodes were relatively uncommon, accounting for only 3% of the total nodes evaluated in our study.

### Discussion

The early detection and accurate staging of malignant nodal disease is important for patient management, but conventional CT and MRI have limited diagnostic performance. Quantitative WB-DWI may improve nodal assessment, but there is paucity of data concerning normal lymph node ADCs.

This study uniquely describes ADC histogram characteristics derived from more than 1000 lymph node volumes of a healthy adult population. The global mean and median ADC of all lymph nodes were 1.12 ± 0.27 and 1.09 (10^− 3^ mm^2^/s), respectively. Most lymph nodes were identified in the cervical, inguinal and axillary region. In prior regional DWI studies, including only the cervical, axillary and inguinal lymph nodes of 13, 16 and 20 healthy volunteers, respectively, the mean ADC of normal lymph nodes ranged between 0.87 and 1.18 (10^− 3^ mm^2^/s) [11, 16, 25]. In another study of pelvic nodes in eight healthy volunteers, the mean ADC was 1.28 (× 10^− 3^ mm^2^/s) [26]. Previous authors noted that normal lymph nodes in the chest and abdomen were poorly depicted on DWI and therefore excluded from analyses [11, 16]. In our study, intrathoracic and some abdominal nodes are also less frequently encountered. The sum of intrathoracic, portal and mesenteric nodes represented only 3% of the total nodes segmented in our study. This may be related to artefacts caused by cardiac and respiratory motion, causing loss of signal and ultimately low visibility on DWI. The significantly higher ADC values and ranges displayed by intrathoracic, portal and retroperitoneal nodes might be attributed to motion of adjacent organs, sampling bias and gradient non-linearity. DWI signal decay caused by the random movement of water molecules can be accentuated by non-linear cardiac or bowel movement, resulting in greater signal decay and consequently overestimation of ADC. As a consequence, nodal ADC of thoracic and some abdominal regions need to be interpreted with caution.

The whole body protocol used in this study follows general clinical recommendations in the choice of b-values, facilitating comparability [27, 28]. The observed measurements lie within mean ADC ranges of previous WB-MRI studies with values of nodes deemed normal on imaging ranging between 1.00 and 1.37 × 10^− 3^ mm^2^/s [11, 13, 29]. Additionally, ADC values were reported to have inter-scanner variability of 14%, accentuated in small volume measurements such as lymph nodes [30]. This may also contribute to measurement differences between centers. It needs to be noted however, that these studies derived “normal “nodal ADCs from patients with underlying malignancy. Hence, these controls may have been subject to reactive changes secondary to treatment or inflammation, which could also affect nodal diffusion characteristics. In contrast, potential confounders in our healthy cohort have been accounted for in the exclusion criteria of this study.

Evaluating more than 1000 lymph nodes allowed us to define the full ADC histogram characteristics of normal lymph nodes. Histogram parameters such as mean, median, skewness and kurtosis were shown to be potential response biomarkers in various types of cancers and lymphoma [12, 13, 31–36]. Consequently, establishing values for normal, healthy conditions is important to allow comparisons also for baseline examinations. One study identified skewness to be a better discriminator between FDG-PET/CT positive and negative lymph nodes in lymphoma patients than mean ADC [13]. In this study, skewness was derived from individual nodes. In contrast, we derived skewness and kurtosis values from the distribution of median individual node ADC values summarized on a per patient or per nodal group basis. Consequently the findings are not directly comparable. Nevertheless, skewness and kurtosis have shown poor repeatability in WB-DWI, and their application requires critical evaluation [37].

Overall, there is large potential for WB-DWI in assessment of nodal disease across the body, as it allows for generation of functional tissue information within short acquisition times, without radioactivity or contrast application. Contemporary literature suggests no significant alteration of T2 relaxation times between normal and diseased nodes, thus making signal and ADC changes dependent on diffusion properties [38]. Knowledge of healthy nodal ADC parameters can improve disease evaluation and is especially useful in morphologically unremarkable nodes. DWI assessment of lymph nodes can become more relevant in the future, as acceleration techniques such as simultaneous multislice DWI acquisition [26] and development of machine learning applications may enhance image quality without increasing acquisition time. Machine learning may also allow fast, automated nodal segmentation and identification of normal nodes and potentially diseased states defined on the basis of ADC thresholds.

This study has limitations, mostly related to the DWI technique. First, misregistration and partial volume effects may degrade ADC assessment of small nodes, the latter was shown to cause up to 12.8% decrease in ADC measurement reproducibility [25]. Second, the ADC may vary over the field of view due to gradient non-linearity [39]. Third, images were acquired on a single scanner, hence immediate application of ADC ranges to other scanners is limited. Nevertheless, the protocol and sequence used are common clinical standard and should allow for general approximation. Fourth, the inter- and intra-observer repeatability of the measurements have not been investigated in this study. Finally, the mean age of 35 years of the volunteers is well below the age of the average cancer patient. Nonetheless, in our limited cohort we could find no significant age dependence of nodal ADCs.

## Conclusions

In conclusion, the global mean and median ADC of all lymph nodes were 1.12 ± 0.27 (10^− 3^ mm^2^/s) and 1.09 (10^− 3^ mm^2^/s). Intrathoracic, portal and retroperitoneal nodes display significantly larger ADCs. Normal intrathoracic, portal and mesenteric nodes are infrequently visualized on WB-DWI of healthy individuals.

## Data Availability

The datasets analysed during the current study are available from the corresponding author on reasonable request.

## Abbreviations

ADC: Apparent diffusion coefficient
DWI: Diffusion-weighted MRI
gADC: Global apparent diffusion coefficient
KDE: Kernel density estimate
TE: Echo time
TR: Repetition time
STIR: Short tau inversion recovery
WB: Whole-body
